# A MEMS ultrasound stimulation system for modulation of neural circuits with high spatial resolution in vitro

**DOI:** 10.1038/s41378-019-0070-5

**Published:** 2019-07-15

**Authors:** Jungpyo Lee, Kyungmin Ko, Hyogeun Shin, Soo-Jin Oh, C. Justin Lee, Namsun Chou, Nakwon Choi, Min Tack Oh, Byung Chul Lee, Seong Chan Jun, Il-Joo Cho

**Affiliations:** 1 Center for BioMicrosystems, Brain Science Institute, Korea Institute of Science and Technology (KIST), Seoul, 02792 Republic of Korea; 2 School of Mechanical Engineering, Yonsei University, Seoul, 03722 Republic of Korea; 3 Division of Bio-Medical Science & Technology, KIST School, Korea University of Science and Technology (UST), Seoul, 02792 Republic of Korea; 4 Center for Neuroscience, Brain Science Institute, Korea Institute of Science and Technology (KIST), Seoul, 02792 Republic of Korea; 5 Convergence Research Center for Diagnosis, Treatment, and Care System of Dementia, Korea Institute of Science and Technology (KIST), Seoul, 02792 Republic of Korea; 6 Center for Glia-Neuron Interaction, Korea Institute of Science and Technology (KIST), Seoul, 02792 Republic of Korea; 7 KU-KIST Graduate School of Converging Science and Technology, Korea University, Seoul, 02841 Republic of Korea; 8 Division of Bio-Medical Science & Technology, KIST School, Korea University of Science and Technology, UST, Yuseong-gu, Daejeon, 34113 Republic of Korea

**Keywords:** Electrical and electronic engineering, Microfluidics

## Abstract

Neuromodulation by ultrasound has recently received attention due to its noninvasive stimulation capability for treating brain diseases. Although there have been several studies related to ultrasonic neuromodulation, these studies have suffered from poor spatial resolution of the ultrasound and low repeatability with a fixed condition caused by conventional and commercialized ultrasound transducers. In addition, the underlying physics and mechanisms of ultrasonic neuromodulation are still unknown. To determine these mechanisms and accurately modulate neural circuits, researchers must have a precisely controllable ultrasound transducer to conduct experiments at the cellular level. Herein, we introduce a new MEMS ultrasound stimulation system for modulating neurons or brain slices with high spatial resolution. The piezoelectric micromachined ultrasonic transducers (pMUTs) with small membranes (sub-mm membranes) generate enough power to stimulate neurons and enable precise modulation of neural circuits. We designed the ultrasound transducer as an array structure to enable localized modulation in the target region. In addition, we integrated a cell culture chamber with the system to make it compatible with conventional cell-based experiments, such as in vitro cell cultures and brain slices. In this work, we successfully demonstrated the functionality of the system by showing that the number of responding cells is proportional to the acoustic intensity of the applied ultrasound. We also demonstrated localized stimulation capability with high spatial resolution by conducting experiments in which cocultured cells responded only around a working transducer.

## Microelectromechanical Systems: Ultrasound stimulation system for modulating neural circuits

A new microelectromechanical system (MEMS) using ultrasound stimulation was able to modulate neurons (nerve cells) or brain slices with high spatial resolution. Neuromodulation (altering nerve activity through targeted stimulation) with ultrasound has attracted attention as a non-invasive approach in treating brain diseases. Hitherto, however, ultrasound neuromodulation has suffered from poor spatial resolution. A team headed by Il-Joo Cho of the Korea Institute of Science and Technology, Republic of Korea developed a MEMS with sufficient power to stimulate neurons and enable precise modulation of neural circuits. The team demonstrated the functionality of the system by showing that the number of responding cells was proportional to the acoustic intensity of the applied ultrasound. The authors believe that their system can serve as a powerful tool in studying the effects of ultrasound on neurons and brain circuits.

## Introduction

Neuromodulation techniques are promising tools for the treatment of brain diseases, such as Parkinson’s disease, epilepsy, and depression^1–3^. Although several neuromodulation methods have been extensively used clinically, they have problems of invasiveness and low spatial resolution. For example, deep brain stimulation (DBS), which is electrical stimulation using electrodes implanted in the deep brain region, improves motor function in patients with Parkinson’s disease^4^, but it requires invasive surgery to implant the electrodes. This technique may entail hazardous problems, such as immune reactions to external materials, infection, and additional surgery for replacement of batteries^5,6^. There are methods that enable neuromodulation through noninvasive brain stimulation, such as transcranial magnetic stimulation (TMS) and transcranial direct current stimulation (tDCS), but these methods provide poor spatial resolution, and they have a low depth of stimulation^7^. As an alternative method for overcoming the problems with the techniques mentioned above, ultrasonic neuromodulation recently has received much attention due to its capability of noninvasive stimulation, especially with its high spatial resolution using low-intensity, focused ultrasound (LIFU)^8^.

Since the possibility of modulating neuronal activity via ultrasound was demonstrated more than 80 years ago, many research groups have assessed the potential of ultrasound for stimulating the brain^9^. Some research groups^10,11^ have worked on developing ultrasonic neuromodulation methods using live animals, while other groups^12,13^ have worked on revealing the underlying mechanism of the ultrasonic neuromodulation with cultured cells or brain slices. Tufail et al. successfully verified that transcranial ultrasound stimulates the intact brain circuits of mice^14,15^. They verified the ultrasonic neuromodulation through the recording of electromyogram (EMG) signals and monitoring the change of muscle contractions followed by stimulating the motor cortex. In addition, Tyler et al.’s in vitro experiments with brain slices of mice showed that ultrasound stimulates neurons and astrocytes^16^. They showed the changes in the activity of neurons and astrocytes modulated by ultrasound through fluorescence imaging of the calcium concentration in cells. Khraiche et al. reported the effect of ultrasound on developing neurons cultured in vitro^17^. They observed that the electrical activity of developing neurons, measured by microelectrode arrays (MEA), increases in response to exposure to ultrasound.

Although many studies have shown the capabilities of ultrasound as a promising tool for neuromodulation^18–22^, the underlying physics and associated mechanisms have remained unknown, and conceivably associated aspects include the effects of acoustic radiation forces on neurons, the roles of neurons and glia, and various types of membrane channels^6,23^. Thus, elaboration of the underlying mechanisms will enable further enhancement or optimization of neuromodulation with ultrasound stimulation^24^. However, to investigate such mechanisms, precise stimulation of brain slices or cells by fine-tuned ultrasound parameters is essential, and it is effective to observe the modulated cells optically because optical observation allows for the monitoring of a larger number of cells for simultaneous recording than electrical recording does in an MEA. Additionally, responses from the stimulated cells must be compared with responses from unstimulated cells to confirm the effect of the ultrasound stimulation. To do so, a localized region must be stimulated with high spatial resolution to enable the precise modulation of neural circuits. However, most previous studies have used commercial ultrasound transducers to stimulate neurons and brain slices. These commercial transducers allow only limited fine adjustment of the distance between the transducers and the cells, so they cannot ensure that the target samples receive accurate acoustic intensity. Additionally, the sizes of the transducers (>tens of millimeters) make it difficult for them to stimulate neurons and brain slices with high spatial resolution. Recently, several miniaturized ultrasound transducers have been developed. Although Lee et al.^25^ and Li et al.^26^ reported miniaturized ultrasound stimulation systems for in vivo applications and showed transcranial ultrasonic neuromodulation by stimulating a specific region that was located several millimeters below the skull, the transducer in these systems still consisted of a large membrane. Kim et al. successfully demonstrated an in vivo experiment with miniaturized ultrasound transducers, but the ultrasound power of a single element was not sufficient for neuromodulation^27^.

**Fig. 1:**
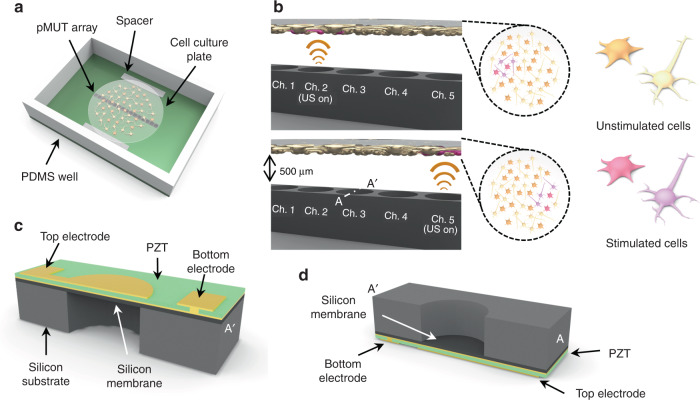
Schematic illustration of the proposed pMUT array for localized ultrasound stimulation. **a** schematic diagram of the pMUT array for ultrasound stimulation on the cell culture plate. A neuron-astrocyte cocultured coverslip located on the pMUT array; **b** conceptual diagram of the localized stimulation showing that cells were stimulated only by ultrasound from an activated transducer; unstimulated cells are bright yellow (left), and stimulated cells are pink (right); **c** cross-section of the bottom view of the pMUT unit; **d** cross-section of the top view of the pMUT unit

Herein, we propose a new microelectromechanical system (MEMS) ultrasound stimulation system for modulating neurons or brain slices with high spatial resolution (Fig. 1). The 16-piezoelectric micromachined ultrasonic transducer (pMUT) array is attached inside a PCB (Printed Circuit Board) and is positioned below the cells or brain slice at a fixed distance to ensure precise control of the intensity of the ultrasound. A polydimethylsiloxane (PDMS)-based fluid chamber for containing cells or brain slices is integrated with the system to provide continuous perfusion of media and delivery of drugs during experiments. This ultrasound stimulation system has three advantages compared with conventional ultrasound transducers used in other reports: (1) ultrasound stimulation with high spatial resolution, (2) accurate control of the intensity of the ultrasound applied on the sample, and (3) compatibility with cell-based experiments. We verified the functionality of the MEMS ultrasound stimulation systems with in vitro experiments by stimulating cocultured neurons and astrocytes. We were able to successfully demonstrate neuromodulation with ultrasound by monitoring calcium transients from the stimulated cells.

## Materials and methods

### Design, fabrication, and packaging of the pMUT array device

To achieve reliable and reproducible localized stimulation, we designed and fabricated an ultrasonic neuromodulation device with a PDMS-based cell culture chamber on a chip (Fig. 1a). The proposed system is composed of an array of 16 ultrasound transducers, a PDMS well for cell culture, and a coverslip where the cells are placed. We integrated the PDMS chamber on the system to make the system compatible with the conventional cell-based experiments with in vitro cultured cells or brain slices. The ultrasound transducer was designed with an array structure to enable localized modulation at the desired stimulation location (Fig. 1b). The distance between transducers and cells was controlled accurately with two spacers to apply reliable power during the experiments.

In the proposed system, we designed the pMUTs to be actuated to generate enough power for the stimulation of cocultured cells at a specific frequency range known to be effective for stimulation^28–30^. The structure of the pMUTs consists of a silicon membrane and a lead zirconate titanate (PZT) layer on the membrane as an actuator (Fig. 1c, d). In the structure, we used a bulk PZT layer for the actuator to acquire enough power for the stimulation. When an electric field is applied to a PZT layer, mechanical deformation occurs due to the unique piezoelectric effects of the material. An alternative signal is applied to cause rapid and repetitive deformation, thereby generating ultrasound^31–33^. In our device, the horizontal deformation of a PZT film was converted to vertical movement of the membrane by attaching the PZT layer on top of the silicon membrane.

We ultimately aimed to utilize our system for in vitro experiments toward revealing the underlying mechanism of ultrasonic neuromodulation in vivo. Therefore, we initially set the resonant frequency of the transducer to 500 kHz, which is known as the reaction frequency of neurons^25–27,31,32^ and a reliable frequency of transcranial ultrasonic neuromodulation in vivo^14^. Then, we determined the dimensions of the pMUTs through a finite element method (FEM) simulation to match the resonant frequency of the pMUTs at 500 kHz. In the simulation, we used the following parameters for the PZT: Young’s modulus of 6.30 × 10^10^ Pa, Poisson’s ratio of 0.34, and density of 7500 kg m^−3^. Our primary purpose was to find an optimal thickness ratio between PZT and silicon to maximize the deflection amplitude of the membrane. The resultant optimized thickness ratio was 0.35, and the corresponding thickness of the PZT layer was 40 μm to minimize the thickness variation after the CMP process of a bulk PZT film. The thickness of the silicon layer was 15 μm. Thinner PZT and silicon layers help to induce greater deflection, but that requires a larger membrane, which makes the spatial resolution poor. Thus, we optimized the dimensions of transducers through the FEM simulations and determined the diameter of 550 μm upon consideration of process variations. The pMUT array was composed of 16 transducers with a center-to-center pitch of 770 μm for accurate modulation of neural circuits in different regions of a sample. The array structure also enabled simultaneous stimulation at different regions in brain slices or cultured cells. In addition, the cells on the unstimulated region can be used as a control group to measure the efficiency of the ultrasound stimulation, which reduces the required number of samples for the experiments.

**Fig. 2:**
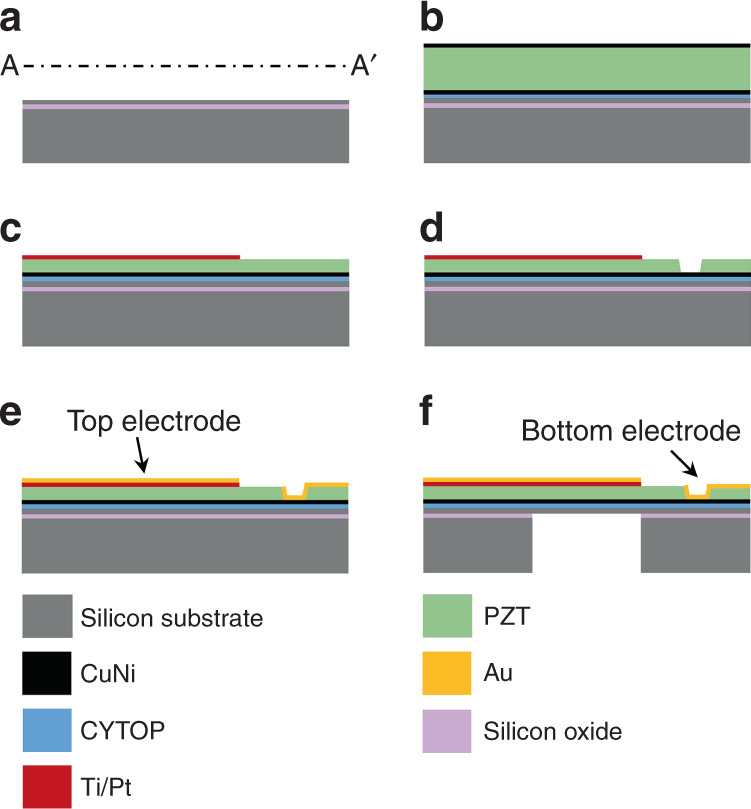
Fabrication process of the pMUT array (section A–A’ in Fig. 1b). **a** A 4-inch silicon-on-insulator (SOI) wafer with 15 μm of top silicon was prepared; **b** the SOI wafer was bonded with a bulk PZT using CYTOP as a bonding layer; **c** the PZT film was thinned using a chemical mechanical polishing (CMP) process; the Ti/Pt layers were deposited and patterned on top of the PZT layer; **d** the PZT layer was etched to form a hole for the bottom electrode; **e** an Au layer was deposited and patterned to form the top and bottom electrodes; **f** the membrane was released from the backside through the DRIE process

**Fig. 3:**
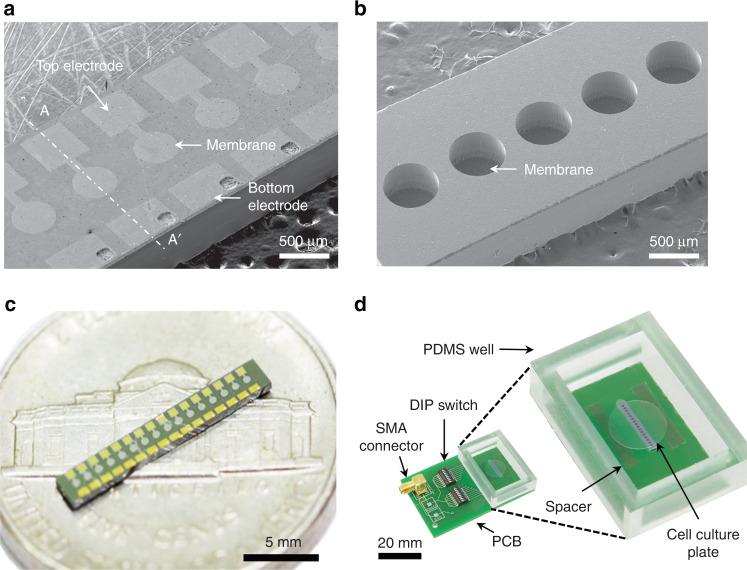
Images of the fabricated pMUT array and packaged device. **a** SEM image of the bottom view of the fabricated pMUT array showing section line (A–A’) of Fig. 2; **b** SEM image of the top view of the fabricated pMUT array; **c** optical image of the fabricated pMUT array; **d** optical image of the packaged device with the pMUT array, showing the DIP switches soldered on the PCB to control individual transducers and spacers to maintain a constant distance between the cell culture plate and the transducers; voltage was applied to the pMUT array through an SMA connector

The proposed pMUT array was fabricated using standard micromachining techniques (Fig. 2). A 4-inch silicon-on-insulator (SOI) wafer was bonded with a 1-mm-thick bulk PZT (PIC 151; PI, Germany) sheet that had CuNi layers as electrodes on both sides. In the proposed process, we spin-coated CYTOP (CTL-809; AGC, UK) on the SOI wafer as a bonding layer. Then, the PZT sheet was bonded on top of the SOI wafer using a wafer bonder at the bonding temperature of 160 °C with a pressure of 3.5 kg·f/cm^2^. Next, we used a chemical mechanical polishing (CMP) process to reduce the thickness of the PZT film to 40 μm. We deposited and patterned 30-nm/300-nm Ti/Pt layers on top of the PZT layer to form the top electrode, which defines the membrane of the transducer. To access the metal layer that was deposited on the PZT layer, we deposited and patterned 150 nm of Cr as a mask layer for etching the PZT layer. Then, we etched the PZT layer using a wet etching process in an H_2_O:HCl:HF (250:10:1) solution, and a hole was formed in the PZT layer. Through the hole, we deposited and patterned a 30-nm/500-nm Cr/Au layer to access the CuNi layer, which was used as a bottom electrode. Finally, we etched the handling wafer from the backside through the DRIE (Deep Reactive Ion Etching) process using an aluminum etch mask to complete the fabrication of the pMUT array (Fig. 3a, b).

The fabricated pMUT array was mounted on the PCB, and electric pads were connected through wire-bonding using gold wires. Dual in-line package (DIP) switches were connected to each transducer for selective control of the transducers (Fig. 3d). Along the pMUT array, we integrated two 500-μm-thick spacers made of PDMS to accurately control the distance between cells and the pMUT array. The PDMS-based cell culture chamber was fabricated separately and integrated with the ultrasound transducer array. We designed the chamber large enough to have the objective lens of the microscope inside the chamber to inspect the cells. The size of the chamber was 42 × 30 × 15 mm, and the thickness of the walls of the chamber was 5 mm. The chamber was attached on the PCB and sealed with glue to prevent any leakage of the cell culture media.

### Measurement of the mechanical characteristics of the ultrasound transducer

**Fig. 4:**
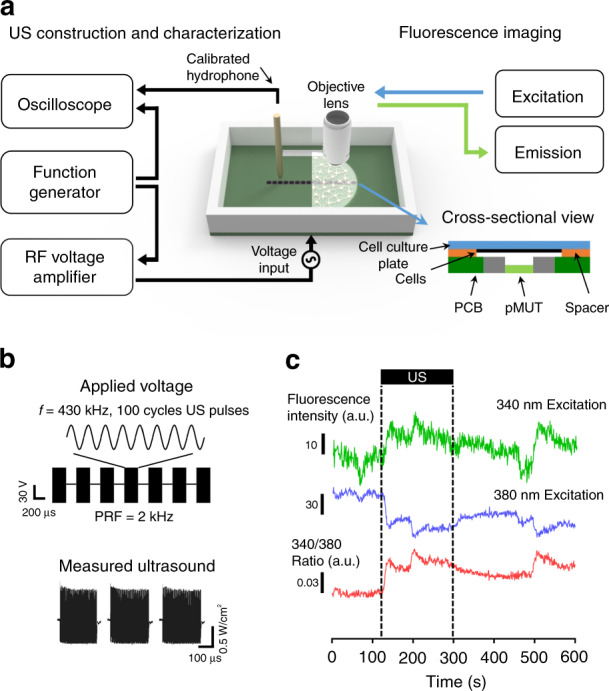
Experimental setup for mechanical characterization and optical recording. **a** schematic diagram of the characterization (left) and general configuration (right) of the system; **b** applied voltage signals of 100 cycles, 430-kHz sine wave pulses to the pMUT array and measured acoustic intensity by a calibrated hydrophone; **c** representative ultrasound-induced fluorescence trace of ratiometric dye Fura-2 AM showing fluorescence intensities that were increased by 340 nm of excitation light and decreased by 380 nm of excitation light

In the ultrasound stimulation of neurons and astrocytes, the frequency and sound pressure of the ultrasound are the most important mechanical parameters. Thus, we measured the mechanical characteristics of the fabricated pMUT array. In this experiment, we filled the chamber with phosphate-buffered saline (PBS) solution to characterize the pMUT device in the liquid phase, which is similar to the environment of cell-based experiments. Then, we used a desiccator to remove the bubbles inside the chamber so that the transducer was immersed completely in the solution during the characterization. When bubbles become trapped in the transducer, its fundamental resonant frequency may be shifted^34,35^, and the ultrasound may be attenuated at the interface between the bubbles and the solution. An impedance/gain-phase analyzer (HP 4294A; Agilent, USA) was used to measure the resonant frequency of the transducer we fabricated. To actuate the pMUTs, we used a function generator (33500B; Agilent, USA) to apply 100 cycles of sinusoidal sound waves with a pulse repetition frequency (PRF) of 2 kHz, which is the effective value for stimulating neurons and astrocytes^14^. However, the actuation voltage of the pMUT array, which was in the range of 20–80 volts, was higher than the voltage generated by the function generator, i.e., 10 V. Thus, we used an RF amplifier (411LA; ENI, USA) to amplify the sinusoidal waves from the function generator with a gain of 40 dB. With the actuation voltage from the RF amplifier, the pMUT array generated the ultrasound, and we measured the pressure level of the sound using a needle hydrophone (NH1000; Precision Acoustics, UK) and a digital oscilloscope (DSO-X 4034 A; Agilent, USA). The tip of the hydrophone was placed 1 mm from the surface of the transducer to measure the acoustic pressure that would be applied to the cells in the in vitro experiments (Fig. 4). All sound pressure was measured based on the ultrasound from a single transducer. The temporal peak acoustic intensity is defined as p^2^/ρc, where p is the ultrasound pressure measured by the hydrophone, ρ is the density of the medium, and c is the velocity of sound in the medium. In this equation, we assumed that the value of ρ was 1000 kg/m^3^ and that c is a velocity of 1480 m/s in water^36,37^.

### Preparation of the in vitro model of brain tissue through a coculture of neurons and astrocytes

We prepared an in vitro model of brain tissue with primary neurons and astrocytes to mimic in vivo brain tissue. To obtain high-purity, high-viability cells, neurons, and astrocytes were dissociated from different rats^38^. The primary astrocytes were dissociated from postnatal day-1 Sprague-Dawley (SD) rats. Embryonic day-18 SD rats were used to isolate the primary cortical neurons to enhance the viability of the cells and the connectivity of the neurites^39^. We used a neural tissue dissociation kit T (MACS; Miltenyi Biotec, USA) to extract astrocytes from the neurons. Next, we prepared a coverslip to be used as a cell culture plate. We cleaned the coverslip with 70% ethanol and sterilized it in an autoclave. To enhance the adhesion of cells to the substrate^40,41^, we coated the sterilized coverslip with Poly-D-lysine (PDL; 100 μg/ml; Sigma-Aldrich, USA) for at least 3 h at 37 °C. Then, we placed the coverslip in the wells of a 24-well plate and filled the well with 500 μl of cell-suspended-culture solution that contained 1 × 10^5^-cells/ml neurons and 0.5 × 10^5^-cells/ml astrocytes. The cell culture medium also contained Neurobasal cell culture medium (Gibco, USA) with 10% fetal bovine serum (Gibco, USA), 2% B27 supplement (Life Technologies, USA), 0.25% Glutamax (Gibco, USA), and 1% penicillin–streptomycin (Gibco, USA). On the following day, we replaced the cell culture medium with a serum-free and antibiotic-free medium. We replaced half of the medium with fresh medium every two days until day 14.

### In vitro experimental procedure

We conducted in vitro experiments to observe the responses of the cells to the ultrasound generated by the transducer array we fabricated. Before the experiment, we sterilized the ultrasound stimulation system with 99% ethanol and rinsed it with distilled water. This was followed by exposing the system to UV light to ensure biocompatibility. Then, we mounted the ultrasound stimulation system on the stage of the microscope to observe the change of cellular activity due to the ultrasound stimulation. In this experiment, we used Fura-2 AM dye to monitor the change in the intracellular concentration of Ca^2+^ and observe the activities of the cells. The representative fluorescence intensity transient of the Fura-2 AM dye showed the change in the intracellular Ca^2+^ concentration of the cultured cells stimulated by our ultrasound transducer (Fig. 4c). Fura-2 AM is a fluorescent indicator of the Ca^2+^ activity that is excitable at either 340 or 380 nm and emits fluorescence at 510 nm. Upon the ultrasound stimulation, we measured fluorescence intensities from cells emitting fluorescence at 510 nm with the sequential excitation at 340 nm (F_340_) and 380 nm (F_380_). Then, the ratiometric fluorescence intensity was estimated by calculating the F_340_/F_380_ ratio. This ratiometric imaging allows for enhanced sensitivity of the changes of Ca^2+^ ions because responding cells emit increased F_340_ and decreased F_380_. The response rate was defined as the number of responding cells divided by the total number of recorded cells.

For loading of Fura-2 AM to neurons and astrocytes, the cells were incubated with 5 μM of Fura-2 AM (Thermo Fisher Scientific, USA) mixed with 1 ml of external solution containing 5 μl of 20% pluronic acid (Thermo Fisher Scientific, USA) for 40 min at room temperature. Then, the ratiometric imaging was performed while stimulating the cells with ultrasound. We attached neuron-astrocyte cocultured samples on the spacer in the PDMS well to maintain a constant distance between the cell culture plate and the transducers. We filled the well with recording solution that contained 10 HEPES, 150 NaCl, 3 KCl, 2 CaCl_2_, 2 MgCl_2_, and 5.5 glucose (in mM), and then we adjusted the pH to 7.3 and the osmolarity to 325 mOsmol kg^−1^. Then, we monitored Ca^2+^ transients for 120 s as a control experiment and turned on the ultrasound transducer for 180 s (from 120 s to 300 s), followed by observing Ca^2+^ transients for 300 s after the transducer was turned off.

### Immunostaining of cocultured neurons and astrocytes

Neuron-astrocyte cocultured samples were fixed with 4% paraformaldehyde phosphate buffer solution (Wako, Japan) to preserve the cytoskeletal structures of cells for 2 h at room temperature. To enhance the permeability of the cell membranes and reduce nonspecific binding of antibodies, we used a blocking solution that contained 0.1% [w/v] Triton X-100 (Sigma-Aldrich, USA) and 2% bovine serum albumin (Sigma-Aldrich, USA) in PBS for 4 h at 4 °C. Next, we stained cells with primary and secondary antibodies for 12 h and 6 h in sequential order at 4 °C. The nuclei were stained with Hoechst 33342 (Molecular Probes, USA) for 30 min at room temperature. We washed the samples three times with PBS for 20 min at room temperature in every step. The primary antibodies that were used were mouse anti-β-tubulin III (Tuj-1; Sigma-Aldrich, USA) and chicken glia fibrillary acidic protein (GFAP; Merck Millipore, USA). Alexa Fluor conjugates (Alexa Fluor 488 and 594; Molecular Probes, USA) were used as secondary antibodies. We obtained fluorescent images of cocultured samples by using an inverted confocal laser scanning microscope (LSM 700; Carl Zeiss, Germany) with solid-state lasers (405, 488, and 555 nm). All immuno-stained images were acquired with a 20× objective (NA 0.3), and they were processed by adjusting fluorescent intensities and merged using ZEN 2012 software (Carl Zeiss, Germany).

## Results and discussion

### Mechanical characteristics of the fabricated pMUTs

**Fig. 5:**
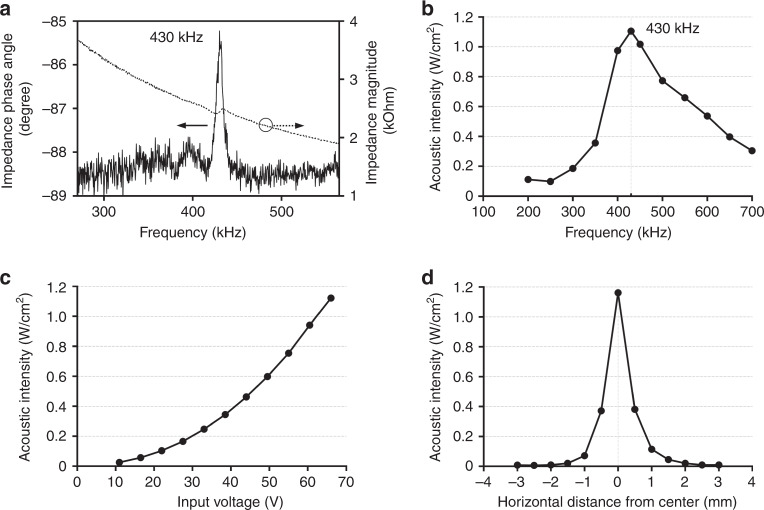
Measured mechanical characteristics of the fabricated pMUTs. **a** Impedance phase angle and magnitude of the fabricated pMUTs at the resonant frequency; **b** acoustic intensity with different frequencies; **c** acoustic intensity with different input voltages; **d** acoustic intensity with different horizontal distances from the center of the transducer; all acoustic intensities were measured by a calibrated hydrophone at a height of 1 mm above the transducer membrane

Before assessing the performance of the pMUTs, we characterized the mechanical characteristics, such as resonant frequency and acoustic intensity. In these experiments, we measured the resonant frequency of the transducer in two different ways. First, we measured an impedance phase angle according to frequencies to find the resonant frequency. Measurements of the impedance phase angle showed that the maximum value was −85.79˚ at 430 kHz (Fig. 5a). The resonant frequency was shown to be a suitable frequency for ultrasound neuromodulation, as reported elsewhere^14,28^. We also measured the resonant frequency of the transducer by measuring the acoustic intensity at various frequencies using a calibrated hydrophone, as shown in the system configuration (Fig. 4a). The peak intensity of 1.122 W/cm^2^ that was measured at 430 kHz with an input voltage of 66 V was identical to the impedance result (Fig. 5b). The 3-dB bandwidth of the transducer was 227.66 kHz measured in a water tank.

At the resonant frequency of 430 kHz, we measured the acoustic intensity with various input voltages to show the range of the ultrasound power that was generated. Additionally, we measured the acoustic intensity according to various horizontal distances from the center of the transducer to verify the localized stimulation. The vertical distance from the transducer to the hydrophone (1 mm) was estimated by the time difference between the signal generated by the function generator and the signal measured by the hydrophone considering the speed of sound. The acoustic intensity was found to range from 0.025 to 1.122 W/cm^2^ when the input voltage of pMUTs was varied from 11 to 66 V (Fig. 5c). This measured acoustic intensity was high enough to perform ultrasonic neuromodulation^28–30^ without causing mechanical or thermal destruction of tissues^42–44^. Additionally, to verify the success of localized stimulation by ultrasound, the acoustic intensity was measured with different horizontal distances from the center of the transducer. Acoustic intensity decreased dramatically as the horizontal distance between the calibrated hydrophone and the center of the transducer increased (Fig. 5d). At a horizontal distance of 1 mm, the acoustic intensity was reduced to less than 10% of its maximum value.

### Neuromodulation by ultrasound on an in vitro platform

**Fig. 6:**
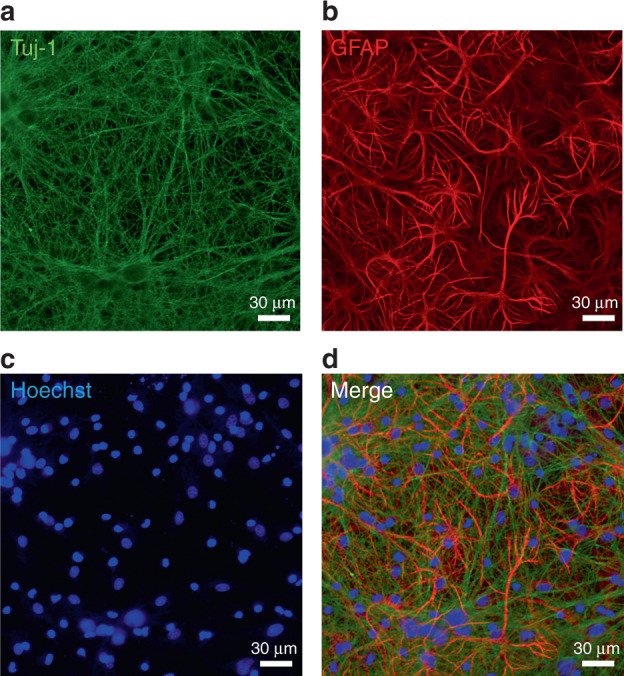
Confocal scanning microscopy Images of neuron-astrocyte cocultured sample at DIV 14. **a** neurons stained by the neuron-specific marker Tuj-1; **b** astrocytes stained by the astrocyte-specific marker, GFAP; **c** nuclei stained by the nuclei-specific marker Hoechst; **d** merged image of cocultured neurons and astrocytes

**Fig. 7:**
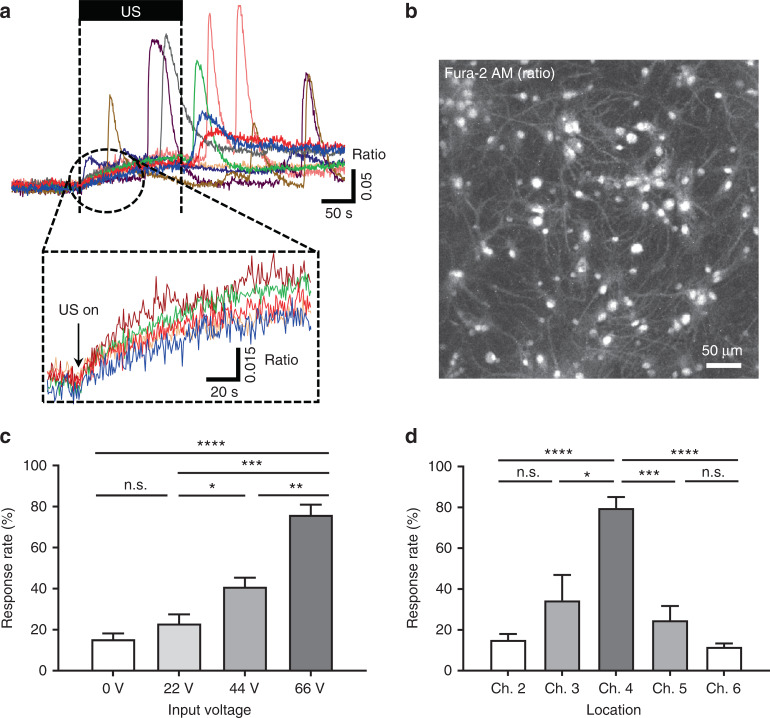
Responses of cocultured cells stimulated by the ultrasound from the pMUT array through measurement of the real-time change in the intracellular Ca^2+^ concentration. **a** plot of the transients in the intracellular concentration change of Ca^2+^ of responding cells by ultrasound at the input voltage of 66 V; magnified view of the plot shows the gradual change of calcium concentration by ultrasound; the different colors represent responses from different cells; **b** fluorescence image of the intracellular calcium-specific marker Fura-2 AM; **c** response rate of stimulated cells by ultrasound with different input voltages; **d** response rate of stimulated cells by ultrasound with different horizontal locations of the Ch. 4 transducer; data are presented as the mean ± SEM. values. Student’s *t*-test were used to analyze the differences. *p < 0.05, **p < 0.01, ***p < 0.001, ****p < 0.0001, n.s: not significant

We wanted to examine the possibility of neuromodulation by ultrasound using our system and with the capability of localized neuromodulation of neural circuits. In this experiment, we exposed an in vitro model of brain tissue to the ultrasound generated by the transducer. We cocultured both neurons and astrocytes for 14 days (Fig. 6). The neurons and astrocytes showed the typical size and morphology of the cell body, and they formed neural networks in a two-dimensional culture. In each experiment, we used a fresh cell plate because, after cells are stimulated by ultrasound, they maintain prolonged excitability after the ultrasound stimulation is turned off. The exact underlying mechanism of the ultrasonic neuromodulation is still unknown. According to hypotheses, a membrane channel, which responded to mechanical stimulation, might be opened by ultrasound and Ca^2+^ ions moving into the cell^23^. As a result, intracellular Ca^2+^ concentration of the cells increases by ultrasound stimulation. We used Fura-2 AM to monitor the real-time calcium transients because its fluorescent intensity changes depending on the concentration of the intracellular Ca^2+^ ^45^. Our device modulated neurons and astrocytes successfully (Fig. 7a); specifically, the Ca^2+^ transients of the cells increased immediately when the ultrasound was turned on. The different colors in the figure represent responses from different cells. Interestingly, the calcium transients remained stimulated even after the ultrasound was turned off. The Ca^2+^ content of the cells that responded to the ultrasound increased immediately when the ultrasound was applied, but no such reaction occurred during or after the exposure in the cells that did not respond to the ultrasound. We were able to categorize the cells that responded to the ultrasound into two types, i.e., cells that showed gradual increases in the calcium concentration and cells that had abrupt increases in the calcium concentration. Additionally, some cells showed changes in the calcium concentration after the ultrasound was turned off, whereas other cells did not. We speculate that these different responses resulted from the specific responses of different types of cells. In other words, neurons and astrocytes responded differently to ultrasound with distinct amplitudes and shapes.

Next, to explore the quantitative effect of the intensity of the ultrasound on cocultured cells, we examined how neurons and astrocytes responded to various ultrasound intensities. We stimulated cells using input voltages for the transducer ranging from 0 to 66 V to modulate the intensity of the ultrasound while keeping the other conditions (i.e., the frequency of the ultrasound and the distance between the transducer and the cells) constant, and we examined the response rate of the cells that were stimulated (Fig. 7c). The average response rate was 15.4 ± 2.83% (*n* = 454, 4 plates) at 0 V. The mean value slightly increased to 23.4 ± 4.37% (*n* = 438, 4 plates) at 22 V, but this result did not show a statistically significant difference from the response rate at 0 V. Cocultured cells with a response rate of 41.0 ± 4.38% (*n* = 422, 4 plates) responded increasingly at 44 V. Expectedly, at 66 V, 76.0 ± 5.00% (*n* = 415, 4 plates) of the cocultured cells responded to the ultrasound. Although some other studies have examined the effects of acoustic intensity on neuromodulation^14,17,29,30,46^, little attention has been paid to the quantitative examination of the response of primary neurons and astrocytes to various ultrasound intensities. For the first time, our experimental results show the quantitative analysis of ultrasonic neuromodulation at the cellular level, which may have an important role in substantiating the underlying mechanisms of neuromodulation by ultrasound and in applying the ultrasound neuromodulation to therapeutic methods for the treatment of brain diseases.

### Localized stimulation with the ultrasound transducer array

We intentionally fabricated a small-sized membrane for the transducer to overcome the disadvantage of poor spatial resolution in commercial transducers. Because the size of the membrane in our device was less than a millimeter, i.e., 550 μm, ultrasound from the transducer could be applied to a localized area, which provided the capability of precisely modulating neuronal circuits in specific regions of the brain. To confirm this localized stimulation, we investigated how the cocultured cells responded according to the horizontal distance from the center of the transducer. When ultrasound was generated by a transducer (Ch. 4), we monitored how many cells located above neighboring transducers (Ch. 2, 3, 4, 5, and 6) responded (Fig. 7d) while keeping the other conditions constant at the input voltage of 66 V. The results showed that 79.7 ± 5.38% (*n* = 509, 5 plates) of the cocultured cells above Ch. 4 responded, which was consistent with the previous results stated above. In the adjacent transducers (Ch. 3 and Ch. 5), 34.5 ± 12.50% (*n* = 504, 5 plates) and 24.7 ± 6.99% (*n* = 484, 5 plates) of the cocultured cells responded, respectively. The response rates of cells above Ch. 3 and 5 (i.e., two channels adjacent to Ch. 4) were statistically insignificant compared with that without the stimulation (response rate of 0 V in Fig. 7c). Finally, in the case of Ch. 2 and Ch. 6, 15.1 ± 2.94% (*n* = 542, 5 plates) and 11.7 ± 1.65% (*n* = 594, 5 plates) of the cocultured cells responded, which was similar to the result at 0 V. Thus, the spatial resolution was almost the size of a single membrane. These data suggest that our ultrasound stimulation system can modulate a localized area successfully, such as specific neural circuits and brain lesions.

Although focused ultrasound requires either a phased array with a complicated control module or an acoustic lens, it has advantages for in vivo applications because of its capability to stimulate a focal spot only. Alternately, direct ultrasound does not require any complicated control module and therefore can be easily applied to in vitro experiments. Thus, it is more useful to use direct ultrasound with a high spatial resolution to examine the underlying mechanism of the ultrasonic neuromodulation. However, the spatial resolution of the proposed system needs to be improved (e.g., to less than 100 μm) while maintaining the current resonant frequency. The reduction of the diameter of the transducer membrane could lead to enhanced spatial resolutions.

Additionally, we can apply this system to in vivo applications by modifying the packaging because the frequency that we used in this study is similar to the frequency widely used for in vivo applications. The lower-frequency ultrasound attenuates less when it transmits through a skull. However, direct ultrasound stimulates not only the target region in the deep brain region but also all of the cells in the location where it passes through tissue. Therefore, we would need to generate a focused ultrasound for localized stimulation of the target region in the brain. Although the optogenetic stimulation allows for extremely high spatial resolution (i.e., individual cellular level), it is an inherently invasive approach that might engender side effects. An electrical stimulation such as DBS is also an invasive technique. In the case of tDCS, it has the advantage of noninvasiveness, but its spatial resolution is poor compared with other neuromodulation techniques. Unlike these techniques, ultrasound is a promising neuromodulation technique due to its noninvasiveness and the relatively high spatial resolution yielded by using a focused ultrasound or MEMS-based ultrasound transducer.

## Conclusions

We fabricated a new pMUT array with a cell culture chamber for modulation of neural circuits by ultrasound with high spatial resolution in vitro. We successfully demonstrated the functionality of the system by showing that the number of responding cells is proportional to the acoustic intensity of the applied ultrasound. We also demonstrated localized stimulation capability with a high spatial resolution through experiments in which cocultured cells responded only around a working transducer. We envision that our proposed device can serve as a powerful tool to study the effect of ultrasound on neurons and brain circuits and to elucidate the underlying mechanism of ultrasonic neuromodulation. Thus, it could be an important advance in our efforts to conquer neurologic diseases.
